# Ameliorating Effect of Various Fractions of *Rumex hastatus* Roots against Hepato- and Testicular Toxicity Caused by CCl_**4**_

**DOI:** 10.1155/2013/325406

**Published:** 2013-05-14

**Authors:** Sumaira Sahreen, Muhammad Rashid Khan, Rahmat Ali Khan

**Affiliations:** ^1^Botanical Sciences Division, Pakistan Museum of Natural History, Garden Avenue, Shakarparian, Islamabad, Pakistan; ^2^Department of Biochemistry, Faculty of Biological Sciences, Quaid-i-Azam University, Islamabad 45320, Pakistan; ^3^Department of Biotechnology, Faculty of Biological Sciences, University of Science and Technology, Bannu, Khyber Pakhtunkhwa 28100, Pakistan

## Abstract

Effect of methanolic extract of *Rumex hastatus* roots (MRR) and its derived fractions, n-hexane (HRR), ethyl acetate (ERR), chloroform (CRR), butanol (BRR), and aqueous extract (ARR), was studied against carbon tetrachloride (CCl_4_) induced hepato and testicular toxicity in rats. Intraperitoneal dose of 20 percent CCl_4_ (0.5 ml/kg bw) was administered twice a week for eight weeks to a group of rats. Other groups were given CCl_4_ and various fractions of *R. hastatus* roots (200 mg/kg bw). CCl_4_ treatment depleted glutathione contents and activities of antioxidant enzymes while increased the concentration of lipid peroxides (TBARS) along with corresponding DNA injuries and histopathological damages. Supplementation with various fractions of *R. hastatus* roots (200 mg/kg body weight) attenuated the toxicity of CCl_4_ in liver and testis tissues through improvement in the serological, enzymatic, and histological parameters towards the normal. Posttreatment of *R. hastatus* roots (200 mg/kg body weight) also reversed the alteration in reproductive hormonal secretions and DNA damages in CCl_4_ treated rats. The results clearly demonstrated that *R. hastatus* treatment augments the antioxidants defense mechanism and provides the evidence that it may have a therapeutic role in free radical mediated diseases.

## 1. Introduction

Reactive oxygen species (ROS), like hydroxyl, peroxyl, and superoxide radicals, are very transient and highly reactive causes of the pathogenesis of atherosclerosis, neurodegeneration, inflammation, cardiovascular diseases, diabetes, and cataracts [1, 2]. CCl_4_ is a toxic chemical, commonly used to induce hepatic cirrhosis [3] and testicular injuries in experimental animals [4]. Metabolic activation of CCl_4_ by cytochrome P_450_ resulted in the production of trichloromethyl radical (^•^CCl_3_) and peroxy trichloromethyl radical (^•^OOCCl_3_) that in turn initiate lipid peroxidation, responsible for injuries in various organs like liver and testis [5]. These free radicals combine with polyunsaturated fatty acids of hepatic and testicular cell membranes, cause elevation of thiobarbituric acid reactive substances (TBARSs) concentration with subsequent necrosis [6], and increase lysosomal enzymes activities [7]. The health promoting effects of antioxidants on oxidative damage are mostly examined through cellular antioxidants enzymes in addition to TBARS and GSH concentration [5, 8]. It is also reported that increase in oxidative damage to sperm membranes, proteins, and DNA is associated with alterations in signal transduction mechanisms that affect fertility [9] and cause degeneration of somniferous tubules showing a relationship between hypogonadism and liver cirrhosis [5]. *Rumex hastatus* D. Don belongs to the Polygonaceae family and is popularly known as “khatimal.” It is distributed in northern Pakistan, northeast Afghanistan, and southwest China, growing between 700 and 2500 m, and sometimes grows as a pure population. It is reported that the whole plant is used as medicine. It is laxative, alterative, tonic, and is used in rheumatism [10] and sexually transmitted diseases including AIDS [11]. Our previous studies substantiated the *R. hastatus* leaves as a good antioxidant source with sufficient amount of phenolics [12]. Zhang et al. [13] by referring the use in Chinese herbal system reported seven phenolic compounds from *R. hastatus* roots. Thus, regarding the cultural/ethnic use present toxicological studies in rat models have been planned to evaluate the protective effect of various fractions of *R. hastatus* roots against hepato- and testicular toxicity caused by CCl_4_.

## 2. Materials and Methods

### 2.1. Extract Preparation

*R. hastatus* District roots were collected from Havelian, Abbottabad, Pakistan. Shade dried roots were powdered in a Willy Mill to 60-mesh size and used for solvent extraction. Five kg powder was extracted twice with 10 liters of 95 percent methanol at 25°C for 48 h and filtered. The methanolic solution was dried in a rotary evaporator (Panchun Scientific Co., Kaohsiung, Taiwan) to obtain methanolic crude extract of *R. hastatus* roots (MRR). In order to resolve the compounds with escalating polarity, a part of the extract was suspended in distilled water and subjected to liquid-liquid partition by using solvents in a sequence of *n*-hexane (HRR), ethyl acetate (ERR), chloroform (CRR), and butanol (BRR), while the remaining soluble portion was filtered and used as aqueous fraction (ARR). After fractioning, the solvent of the respective fraction was evaporated by rotary evaporator [14].

### 2.2. *In Vivo* Evaluation of Fractions

For *in vivo* studies six-week-old male Sprague-Dawley rats weighting 180 ± 10 g were provided with food and water *ad libitum* and kept at 20–22°C on a 12 h light-dark cycle. All experimental procedures involving animals were conducted in accordance with the guidelines of National Institutes of Health, Islamabad, Pakistan. The study protocol was approved by Ethical Committee of Quaid-i-Azam University, Islamabad Pakistan. The rats were acclimatized to laboratory condition for 7 days before commencement of experiments.

### 2.3. Experimental Plan

For subchronic toxicity, eight-week experiment was designed according to Shyu et al. [3]. Ninety-six rats were randomly divided into sixteen groups (6 rats of each group). Group I animals remained untreated, while Group II animals received olive oil and DMSO twice a week for eight weeks. Animals of Groups III, IV, V, VI, VII, VIII, IX, and X received intraperitoneally 0.5 mL of CCl_4_, (20 percent in olive oil) twice a week for eight weeks. Group III received only CCl_4_, while Group IV administered silymarin at a dose of 50 mg/kg bw after 48 h of CCl_4_ treatment. Groups V, VI, VII, VIII, IX, and X received different fractions at a dose of 200 mg/kg bw, HFC, EFC, CFC, BFC, MFC, and AFC, twice a week for eight weeks orally. However, Groups XI, XII, XIII, XIV, XV, and XVI received fractions (200 mg/kg bw) alone twice a week for eight weeks after 48 hr of CCl_4_ treatment orally. At the end of eight weeks, after 24 h of the last treatment, animals were given chloroform anesthesia and dissected from ventral side. Blood was drawn and centrifuged at 1500 ×g for 10 min, at 4°C to collect the serum. Liver and testis tissues were perfused with ice cold saline and excised. Subsequently, half of both tissue portions were treated with liquid nitrogen and stored at −80°C for further enzymatic and DNA damage analysis, while other portions were processed for histology.

### 2.4. Analysis of Serum

For estimation of liver function tests serum samples were assayed for ALT, AST, ALP,-*γ*-GT, total cholesterol, triglycerides, LDL, and HDL by using standard AMP diagnostic kits (Graz, Austria).

Serum analysis of testicular hormones like FSH, LH, testosterone, prolactin, and estradiol was radioimmunoassayed by using Marseille Cedex 9 France Kits and Czech Republic Kits from Immunotech Company.

### 2.5. Assessment of Antioxidant Enzymes

Ten percent of homogenates of liver and testis tissues were prepared separately in 100 mM KH_2_PO_4_ buffer containing 1 mM EDTA (pH 7.4) and centrifuged at 12,000 ×g for 30 min at 4°C. The supernatant was collected and used for the following experiments as described below. Protein concentration of the supernatant was determined by the method of Lowry et al. [15] using crystalline bovine serum albumin as standard.

#### 2.5.1. Catalase Assay (CAT)

CAT activities were determined by using H_2_O_2_ as a substrate [16]. 0.1 mL of the supernatant was mixed with 2.5 mL of 50 mM phosphate buffer (pH 5.0) and 0.4 mL of 5.9 mM H_2_O_2_, and change in absorbance was recorded at 240 nm after one min. One unit of CAT activity was defined as an absorbance change of 0.01 as units/min.

#### 2.5.2. Peroxidase Assay (POD)

In this method guaiacol was used as the substrate [16]. For the POD activity determination 0.1 mL of the supernatant was added to the reaction mixture having 2.5 mL of 50 mM phosphate buffer (pH 5.0), 0.1 mL of 20 mM guaiacol, and 0.3 mL of 40 mM H_2_O_2_. Changes in absorbance of the reaction solution at 470 nm were determined after one min. One unit of POD activity was defined as an absorbance change of 0.01 units/min.

#### 2.5.3. Superoxide Dismutase Assay (SOD)

In this method NADH was used as the substrate [17]. Reaction mixture of this method contained 0.1 mL of phenazine methosulphate (186 *μ*M), 1.2 mL of sodium pyrophosphate buffer (0.052 mM; pH 7.0), and 0.3 mL of supernatant after centrifugation (1500 ×g for 10 min followed by 10000 ×g for 15 min) of tissue homogenate was added to the reaction mixture. Enzyme reaction was initiated by adding 0.2 mL of NADH (780 *μ*M) and stopped after 1 min by adding 1 mL of glacial acetic acid. Amount of chromogen formed was measured by recording color intensity at 560 nm. Results are expressed in units/mg protein.

#### 2.5.4. Glutathione-S-Transferase Assay (GST)

Glutathione-S-transferase activity was assayed by the method of Habig et al. [18]. The reaction mixture consisted of 1.475 mL phosphate buffer (0.1 mol, pH 6.5), 0.2 mL reduced glutathione (1 mM), 0.025 mL of 1-chloro-2,4-dinitrobenzene (CDNB) (1 mM), and 0.3 mL of supernatant in a total volume of 2.0 mL. The changes in the absorbance were recorded at 340 nm, and enzymes activity was calculated as nM CDNB conjugate formed/min/mg protein using a molar extinction coefficient of 9.6 × 10^3^ M^−1^ cm^−1^.

#### 2.5.5. Glutathione Reductase Assay (GSR)

Glutathione reductase activity was determined by the method of Carlberg and Mannervik [19]. The reaction mixture consisted of 1.65 mL phosphate buffer (0.1 mol; pH 7.6), 0.1 mL EDTA (0.5 mM), 0.05 mL oxidized glutathione (1 mM), 0.1 mL NADPH (0.1 mmol), and 0.1 mL of supernatant in a total volume of 2 mL. Enzyme activity was quantitated at 25°C by measuring disappearance of NADPH at 340 nm and was calculated as nM NADPH oxidized/min/mg protein using molar extinction coefficient of 6.22 × 10^3^ M^−1^ cm^−1^.

#### 2.5.6. Glutathione Peroxidase Assay (GSH-Px)

Glutathione peroxidase activity was assayed by the method of Mohandas et al. [20]. The reaction mixture consisted of 1.49 mL phosphate buffer (0.1 M; pH 7.4), 0.1 mL EDTA (1 mM), 0.1 mL sodium azide (1 mM), 0.05 mL glutathione reductase (1 IU/mL), 0.05 mL GSH (1 mM), 0.1 mL NADPH (0.2 mM), 0.01 mL H_2_O_2_ (0.25 mM), and 0.1 mL of supernatant in a total volume of 2 mL. The disappearance of NADPH at 340 nm was recorded at 25°C. Enzyme activity was calculated as nM NADPH oxidized/min/mg protein using molar extinction coefficient of 6.22 × 10^3^ M^−1^ cm^−1^.

#### 2.5.7. Quinone Reductase Assay (QR)

The activity of quinone reductase was determined by the method of Benson et al. [21]. The 3.0 mL reaction mixture consisted of 2.13 mL Tris-HCl buffer (25 mM; pH 7.4), 0.7 mL BSA, 0.1 mL FAD, 0.02 mL NADPH (0.1 mM), and 0.l mL of supernatant. The reduction of dichlorophenolindophenol (DCPIP) was recorded at 600 nm, and enzyme activity was calculated as nM of DCPIP reduced/min/mg protein using molar extinction coefficient of 2.1 × 10^4^ M^−1^ cm^−1^.

### 2.6. Reduced Glutathione Assay (GSH)

Reduced glutathione was estimated by the method of Jollow et al. [22]. 1.0 mL sample of supernatant was precipitated with 1.0 mL of (4 percent) sulfosalicylic acid. The samples were kept at 4°C for 1 h and then centrifuged at 1200 ×g for 20 min at 4°C. The total volume of 3.0 mL assay mixture contained 0.1 mL filtered aliquot, 2.7 mL phosphate buffer (0.1 M; pH 7.4), and 0.2 mL of 1,2-dithio-bis-nitrobenzoic acid DTNB (100 mM). The yellow color developed was read immediately at 412 nm on a SmartSpec Plus Spectrophotometer. It was expressed as *μ*M GSH/g tissue.

### 2.7. Estimation of Lipid Peroxidation (TBARS)

The assay for lipid peroxidation was carried out following the modified method of Iqbal et al. [23]. One milliliter of 20 percent TCA aqueous solution and 1.0 mL of 0.67 percent TBA aqueous solution was added to 0.6 mL of phosphate buffer (0.1 M; pH 7.4) and 0.4 mL of homogenate sample. The reaction mixture was heated in a boiling water bath for 20 min and then shifted to crushed ice-bath before centrifuging at 2500 ×g for 10 min. The amount of TBARS formed in each of the samples was assessed by measuring optical density of the supernatant at 535 nm using spectrophotometer against a reagent blank. The results were expressed as nM TBARS/min/mg tissue at 37°C using molar extinction coefficient of 1.56 × 10^5^ M^−1^ cm^−1^.

### 2.8. Hydrogen Peroxide Assay (H_2_O_2_)

Hydrogen peroxide (H_2_O_2_) was assayed by H_2_O_2_-mediated horseradish peroxidase-dependent oxidation of phenol red by the method of Pick and Keisari [24]. 2.0 mL of homogenate sample was suspended in 1.0 mL of solution containing phenol red (0.28 nM), horse radish peroxidase (8.5 units), dextrose (5.5 nM), and phosphate buffer (0.05 M; pH 7.0) and was incubated at 37°C for 60 min. The reaction was stopped by the addition of 0.01 mL of NaOH (10 N) and then centrifuged at 800 ×g for 5 min. The absorbance of the supernatant was recorded at 610 nm against a reagent blank. The quantity of H_2_O_2_ produced was expressed as nM H_2_O_2_/min/mg tissue based on the standard curve of H_2_O_2_ oxidized phenol red.

### 2.9. DNA Fragmentation Assay

DNA fragmentation assay was conducted using the procedure of Wu et al. [25]. Tissue samples (50 mg) were homogenized in 10 volumes of a TE solution pH 8.0 (5 mM Tris-HCl, 20 mmol EDTA) and 0.2 percent triton X-100. 1.0 mL aliquot of each sample was centrifuged at 27,000 ×g for 20 min to separate the intact chromatin (pellet, B) from the fragmented DNA (supernatant, T). The pellet and supernatant fractions were assayed for DNA content using a freshly prepared DPA (Diphenylamine) solution for reaction. Optical density was read at 620 nm at (SmartSpec Plus Spectrophotometer catalog no. 170-2525) spectrophotometer. The results were expressed as an amount of percent fragmented DNA by the following formula:

(1) $$\begin{array}{c} \;\text{percent}\;\;\text{fragmented}\;\;\text{DNA}=\text{T}\times \frac{100}{\text{T}+\text{B}}. \end{array}$$

### 2.10. DNA Ladder Assay

DNA was isolated from tissue samples by using the method of Wu et al. [25] to estimate DNA damages. 5 *μ*g of DNA of rats separately was loaded in 1.5 percent agarose gel containing 1.0 *μ*g/mL ethidium bromide including DNA standards (0.5 *μ*g per well). After electrophoresis gel was studied under gel doc system and was photographed through digital camera.

### 2.11. Histopathological Studies

For microscopic evaluation tissues were fixed in a fixative (absolute alcohol 60 percent, formaldehyde 30 percent, glacial acetic acid 10 percent) and embedded in paraffin, sectioned at 4 *μ*m, and subsequently stained with hematoxylin and eosin. Sections were studied under light microscope (DIALUX 20 EB) at 10x magnifications. Slides of all the treated groups were studied and photographed.

### 2.12. Statistical Analysis

Data are expressed as means ± SD (*n* = 6), and significant differences between the groups were statistically analyzed by Duncan's multiple range test (Statistica Software, 1990). Concentration of significance among the various treatments was determined at *P* < 0.05. 

## 3. Result

### 3.1. Effects of *R. hastatus* Roots on Liver Function Test and Biochemical Markers

The serological concentrations of AST, ALT, ALP, and *γ*-GT are highly susceptible to oxidative stress in liver tissue as shown in Table 1. Chronic CCl_4_ treatment considerably (*P* < 0.05) augmented the concentrations of serum marker enzymes of liver which was attenuated significantly (*P* < 0.05) by oral administration of various fraction of *R. hastatus* roots. However, various fractions of *R. hastatus* roots alone showed the same serum enzyme concentration like that of control group. Hepatotoxin also reacts with polyunsaturated fatty acids to cause lipid peroxidation by disturbing lipid profile as summarized in Table 2. These parameters were significantly restored (*P* < 0.05) by various fractions of *R. hastatus* roots near to control. For serological investigations fractions can be ordered as BRR > MRR > ARR > CRR > ERR > HRR.

### 3.2. Effects of* R. hastatus *Roots on Male Reproductive Hormones of Rats

To estimate the testicular toxicity, reproductive hormones act as effective biomarkers. CCl_4_ intoxication alters the secretion of pituitary and reproductive hormonal concentration. The effects of various fractions of *R. hastatus *roots against CCl_4_ toxicity on hormonal concentration of testosterone, luteinizing hormone (LH), follicle stimulating hormone (FSH), prolactin, and estradiol are summarized in Table 3. CCl_4_ intoxicated rats considerably (*P* < 0.05) decreased the testosterone, FSH, and LH concentration of serum while significantly (*P* < 0.05) raised the prolactin and estradiol concentration. The serum concentrations of LH, testosterone, prolactin, and estradiol were restored (*P* < 0.05) by oral administration of various fractions of *R. hastatus *roots near to control group except HRR and ERR that showed no significance for luteinizing hormone.

### 3.3. Effects of* R. hastatus *Roots on Testis Enzymatic Antioxidant Concentrations

In the present study scavenging effects of various antioxidant enzymes were assessed. The effects of various fractions of* R. hastatus *roots against CCl_4_ intoxication on tissue soluble protein and antioxidant enzyme system such as CAT, POD, SOD, TBARS, and H_2_O_2_ testis are reported in Table 4. Free radicals generated by CCl_4_ injection, disturbing the cell membrane by reacting with phospholipids, leading to lipid peroxidation, caused significant elevation of TBARS and H_2_O_2_ content. Our results point to the ability of CCl_4_ on tissue to cause significant damage by decreasing the tissue protein as well as CAT, POD, and SOD activities in addition to increasing the lipid peroxidation and hydrogen peroxide contents versus control group. Posttreatment of various fractions of* R. hastatus *roots with CCl_4_ improved the activity of reduced enzymes and the soluble protein whereas reduced the concentration of TBARS and H_2_O_2_. The effects of various fractions of *R. hastatus *roots on phase II antioxidant enzymes like GST, GPx, GR, GSH, QR, and DNA fragmentation percent of testicular tissue are presented in Table 5. Administration of CCl_4_ significantly (*P* < 0.05) decreased the glutathione status of GST, GPx, GR, GSH, and QR while amplifying the percent fragmentation of DNA. Posttreatment of various fractions of *R. hastatus *roots along with CCl_4_ treatment markedly improved the activities of GST, GPx, GR, GSH, QR, and percent DNA fragmentation.

### 3.4. Effects of *R. hastatus* Roots on DNA Damages (Ladder Assay)

**Figure 1:**
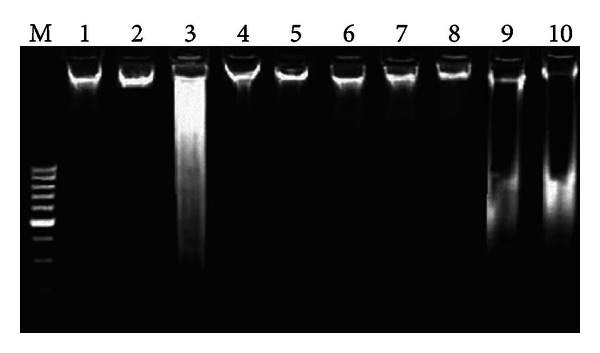
Agarose gel showing DNA damage by CCl_4_ and protective effects of various fractions of *R. hastatus* leaves in testicular tissue. Lanes from left (M) low molecular weight marker, (1) control, (2) DMSO + olive oil group, (3) CCl_4_ group, (4) silymarin + CCl_4_ group, (5) MRR + CCl_4_ group, (6) BRR + CCl_4_ group, (7) ARR + CCl_4_ group, (8) CRR + CCl_4_ group, (9) ERR + CCl_4_ group, and (10) HRR + CCl_4_ group.

CCl_4_ induces DNA damages in the testicular tissues of rats. DNA ladder assay showed that intact genomic DNA was found in control as well as DMSO treated group. Conversely, CCl_4_ group showed severe DNA damages. Postadministration of silymarin and different fractions of *R. hastatus *roots showed reduction in DNA damages as DNA band patterns of these groups were more similar to control group (Figure 1).

### 3.5. Effects of* R. hastatus *Roots on Testis Histoarchitecture

**Figure 2:**
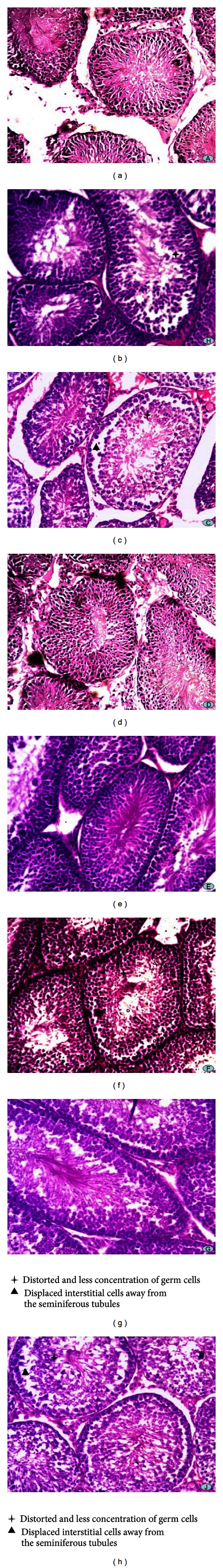
Microphotograph of rat testis (H&E stain). (a) Representative section of testis from the control group showing normal histology, (b) CCl_4_ group, (c) MRR + CCl_4_ group, (d) BRR + CCl_4_ group, (e) ARR + CCl_4_ group, (f) CRR + CCl_4_ group, (g) ERR + CCl_4_ group, and (h) HRR + CCl_4_ group.

Figure 2 illustrates the histological examination of testicular tissues of different treatment groups. Microscopic assessment of male reproductive system revealed the normal seminiferous tubules, sperms with normal morphology, and concentration in control as shown in Figure 2(a). Histological structure of germ cells was found to be normal in appearance.  Figure 2(b) demonstrates that CCl_4_ intoxication caused degenerative changes such as loss of germ cells, abnormality of germinative epithelium, interruption in meiosis, sperm with abnormal shape and concentration, and delocalization of seminiferous tubules. These changes were markedly reduced with oral administration of various fractions of *R. hastatus *roots or silymarin revealing a marked repairing of testicular abnormalities. Among all the tested samples of *R. hastatus *roots, MRR and BRR as shown in Figures 2(c) and 2(d) demonstrated maximum antioxidant and healing effects against CCl_4_ induced damage showing sperm with normal morphology and concentration near to control group. Histopathological findings are in accord with the results of above studied parameters for testicular toxicity.

### 3.6. Effects of *R. hastatus* Roots on Histopathology of Liver

**Figure 3:**
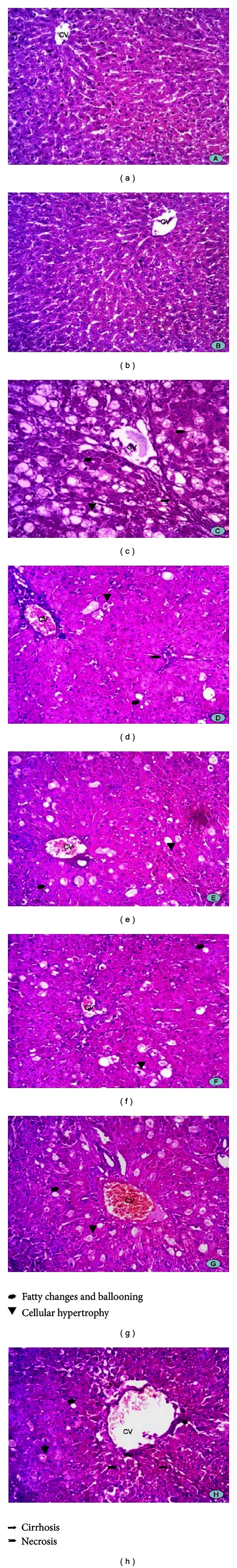
Microphotograph of rat liver (H&E stain). (a) Representative section of liver from the control group showing normal histology, (b) CCl4 group, (c) MRR + CCl_4_ group, (d) BRR + CCl_4_ group, (e) ARR + CCl_4_ group, (f) CRR + CCl_4_ group, (g) ERR + CCl_4_ group, and (h) HRR + CCl_4_ group.

Slides of liver tissues were prepared for histopathological study and stained with hematoxylin and eosins as shown in Figure 3.  Figure 3(c) depicts that administration of CCl_4_ causes fatty changes like ballooning of cells, inflammatory cells infiltrations, dilation of central vein, cellular hypertrophy, necrosis, and degeneration of the lobular architecture. CCl_4_ administration for eight weeks resulted in chronic injury in the form of hepatic cirrhosis. Postadministration of various fractions of *R. hastatus* roots attenuated the hepatic injuries and percent them such as with very less or no fatty changes, no dilation of blood vessel, and uniform morphology of hepatocytes near to control group as shown in Figures 3(c)–3(h). Abnormal changes were not found in the morphology of control group (Figure 3(a)).

## 4. Discussion 

Medicinal plants extracts and their bioactive metabolites play important role in the prevention of oxidative damages especially CCl_4_ induced hepatic and testicular injuries in experimental animals [7, 26]. In the present investigations administration of various fractions of *R. hastatus* revealed reduction in elevated concentrations of AST, ALT, ALP, and *γ*GT to maintain the structural consistency of the hepatocellular structure. Our findings are in agreement with Singh et al. [6] who reported that rise in serum markers has association with immense centrilobular necrosis, cellular infiltration, and ballooning of liver. CCl_4_ treatment caused alteration in cholesterol profile which was significantly reversed with postadministration of various fractions of *R. hastatus*. Similar investigations were reported by Wang et al. [27] while working on female rats to assess hepatic protection of Noni fruit juice against CCl_4_ induced chronic liver damage. Like testicular histology, serum gonadotropin releasing hormone (GnRH) including LH and FSH concentrations may facilitate in discovering conclusion about toxicosis. The reduction in serum testosterone concentrations indicates either a direct effect of chemical (CCl_4_) at Leydig cell concentration or an indirect effect by disturbing the hormonal environment at hypothalamopituitary axis [28] due to oxidative trauma in CCl_4_ treated rats. Tohda et al. [29] also reported that abnormal concentration of intratesticular testosterones inhibits spermatogenesis. The production of testosterone in Leydig cells is stimulated by LH, which activates FSH to bind with Sertoli cells to stimulate spermatogenesis [30]. CCl_4_ insults revealed the suppression in FSH concentration of serum that was in consistency with Khan and Ahmed [5] who reported significant reduction in serum FSH concentration. CCl_4_ intoxicated rats show the malfunctioning of pituitary to secrete FSH and LH indicating that testicular dysfunction leads to infertility. Estradiol directly stimulates the pituitary by determining prolactinemia, with hypothalamic dysfunction in case of hypogonadism. Thus, increased concentration of estradiol and prolactin may also be liable for the origin of hypogonadism in the present study. GSH concentrations are dependent upon the activities of glutathione reductase (GR) and NADH [31]. Glutathione system including GPx, GR, GST, as well as SOD and CAT represents a mutually loyal team of defense against ROS [32]. Enhanced lipid peroxidations expressed in terms of TBARS determine structural and functional alterations of cellular membranes [33]. In the present study, administration of various fractions of *R. hastatus* improved the activities of antioxidant enzymatic (SOD, CAT, POD, GPx, GST, GR, and QR) as well as nonenzymatic (GSH, TBARS, and H_2_O_2_). Hence, the present results regarding chronic toxicity of CCl_4_ are in accordance with previous reports of Khan and Ahmed [5] while studying the protective effects of *Digera muricata* (L.) Mart on testis against CCl_4_ oxidative stress. It was reported that CCl_4_ resulted in the oxidative damage to testicular proteins and DNA in rats [4, 34]. From the present study, it can be assumed that various fractions of *R. hastatus* ameliorated the toxic effects on DNA as revealed by percent DNA fragmentation and ladder assay. The present study clearly augments the defensive mechanism of various samples against oxidative stress induced by CCl_4_ and provides confirmation about its therapeutic use in reproductive abnormalities. Hepatohistology of CCl_4_ intoxicated rats revealed necrosis, fatty changes, cellular hypertrophy, infiltrated kupffer cells and lymphocyte, cirrhosis, and nuclear degeneration in some areas, which was markedly diminished by induction of various fractions of *R. hastatus*. Our study revealed similar investigation which is in agreement with earlier findings [3], while evaluating the medicinal activity of plants against CCl_4_ stimulated hepatotoxicity in rats. The CCl_4_ challenge revealed testicular destruction [35] and degeneration in histological architecture like that of profenofos that was recorded by Moustafa et al. [36]. Data of the present study revealed that CCl_4_ may cause proliferative behavior of testicular cells and obstruct reproduction. However, groups administered various fractions of *R. hastatus* demonstrated a quality active spermatogenesis, thin basement membranes, and normal seminiferous tubules in most of the part of testis. Same histopathology was noticed by Manjrekar et al. [37] while evaluating the protective effects of *Phyllanthus niruri* Linn. on testis against CCl_4_ intoxication.

## 5. Conclusions

It can be concluded from the current study that various fractions of *R. hastatus* roots have the ability to recover the metabolic enzymatic activities and repair cellular injuries, thus providing scientific evidence in favour of its pharmacological use in hepatic and testicular dysfunctioning.

## Figures and Tables

**Table 1:** Effects of various fractions of *R. hastatus* roots on liver function tests.

Group	AST (U/L)	ALT (U/L)	ALP (U/L)	*γ*-GT (U/L)
Control	78.25 ± 2.09^++^	65.23 ± 1.78^++^	121.65 ± 2.19^++^	1.94 ± 0.07^++^
Oil + DMSO	76.46 ± 2.94^++^	64.74 ± 2.02^++^	123.29 ± 2.55^++^	1.95 ± 0.16^++^
CCl_4_	240.15 ± 4.19**	198.93 ± 4.27**	350.79 ± 5.31**	5.11 ± 0.53**
Silymarin + CCl_4_	104.23 ± 3.24^++^	89.28 ± 2.19^++^	171.54 ± 2.63^++^	2.27 ± 0.13^++^
HRR + CCl_4_	213.23 ± 4.21^+^	130.94 ± 4.18^++^	235.67 ± 4.86^++^	3.15 ± 0.48^++^
ERR + CCl_4_	202.18 ± 5.23^+^	128.32 ± 3.15^++^	229.92 ± 5.95^++^	3.01 ± 0.36^++^
CRR + CCl_4_	176.33 ± 3.09^++^	106.23 ± 2.56^++^	207.11 ± 4.23^++^	2.79 ± 0.11^++^
BRR + CCl_4_	142.01 ± 3.29^++^	94.28 ± 2.66^++^	189.61 ± 2.16^++^	2.72 ± 0.13^++^
MRR + CCl_4_	139.28 ± 4.13^++^	99.91 ± 2.73^++^	193.26 ± 3.42^++^	2.57 ± 0.32^++^
ARR + CCl_4_	170.45 ± 4.27^++^	90.34 ± 3.11^++^	175.24 ± 3.18^++^	2.33 ± 0.12^++^
HRR alone	78.45 ± 1.29^++^	63.26 ± 1.45^++^	124.65 ± 1.27^++^	1.93 ± 0.10^++^
ERR alone	75.37 ± 1.55^++^	65.34 ± 1.34^++^	122.15 ± 2.67^++^	1.90 ± 0.09^++^
CRR alone	79.43 ± 0.95^++^	68.86 ± 0.93^++^	123.15 ± 1.23^++^	1.92 ± 0.17^++^
BRR alone	76.76 ± 1.56^++^	66.56 ± 2.20^++^	120.55 ± 2.32^++^	1.99 ± 0.04^++^
MRR alone	74.51 ± 1.34^++^	62.02 ± 0.74^++^	125.34 ± 1.22^++^	1.97 ± 0.07^++^
ARR alone	77.45 ± 1.75^++^	63.36 ± 1.34^++^	122.20 ± 2.85^++^	2.01 ± 0.07^++^

Mean ± SE (*n* = 6 number).

**indicate significance from the control group at *P* < 0.01 probability level.

^++^indicate significance from the CCl_4_ group at *P* < 0.01 probability level.

**Table 2:** Effects of various fractions of *R. hastatus* roots on lipid profile.

Group	Triglycerides (mg/dL)	Total cholesterol (mg/dL)	HDL (mg/dL)	LDL (mg/dL)
Control	140.00 ± 3.57^e^	29.14 ± 1.59^d^	41.23 ± 1.44^d^	23.25 ± 1.01^d^
Oil + DMSO	139.89 ± 4.73^e^	30.00 ± 1.34^d^	40.92 ± 1.71^d^	23.98 ± 1.26^d^
CCl_4_	263.67 ± 2.62^a^	72.09 ± 1.99^a^	60.80 ± 2.89^a^	37.21 ± 1.98^a^
Sily + CCl_4_	182.14 ± 2.36^d^	40.80 ± 2.18^c^	47.29 ± 1.70^c^	27.83 ± 1.71^c^
HRR + CCl_4_	205.23 ± 3.17^c^	64.30 ± 1.79^b^	56.36 ± 0.77^b^	34.38 ± 0.16^b^
ERR + CCl_4_	213.85 ± 3.25^b^	60.96 ± 2.64^b^	54.50 ± 1.24^b^	33.65 ± 0.84^b^
CRR + CCl_4_	199.13 ± 4.44^c^	56.36 ± 3.60^b^	50.97 ± 1.12^c^	30.35 ± 1.14^c^
BRR + CCl_4_	189.16 ± 3.82^c^	42.51 ± 2.84^c^	46.54 ± 1.56^c^	27.93 ± 1.28^c^
MRR + CCl_4_	194.83 ± 2.92^c^	44.89 ± 2.06^c^	45.27 ± 1.46^c^	29.51 ± 2.08^c^
ARR + CCl_4_	190.65 ± 3.54^c^	54.57 ± 2.35^b^	49.35 ± 0.43^c^	31.23 ± 0.66^c^
HRR alone	138.45 ± 1.35^e^	31.55 ± 1.44^d^	40.56 ± 1.33^d^	24.78 ± 0.15^d^
ERR alone	137.96 ± 2.50^e^	32.14 ± 1.32^d^	41.66 ± 1.45^d^	23.25 ± 1.35^d^
CRR alone	140.26 ± 1.64^e^	30.23 ± 1.74^d^	42.55 ± 1.56^d^	22.98 ± 1.01^d^
BRR alone	138.76 ± 1.41^e^	28.15 ± 1.46^d^	40.12 ± 1.68^d^	24.98 ± 0.23^d^
MRR alone	141.01 ± 1.62^e^	33.10 ± 2.81^d^	42.20 ± 0.72^d^	22.25 ± 0.95^d^
ARR alone	139.78 ± 3.84^e^	29.14 ± 1.36^d^	39.23 ± 1.45^d^	23.25 ± 0.16^d^

Values are mean ± SD (06 number). Sily: Silymarin.

^a–d^(means with different letters) indicate significance at *P* < 0.05.

**Table 3:** Effects of various fractions of *R. hastatus* roots on male reproductive hormonal concentration.

Group	Testosterone (ng/mL)	Luteinizing hormone (ng/mL)	Follicle stimulating hormone (ng/mL)	Prolactin (ng/mL)	Estradiol (ng/mL)
Control	2.87 ± 0.09^h^	3.08 ± 0.06^d^	45.63 ± 0.27^h^	10.23 ± 1.41^f^	15.23 ± 0.78^g^
Oil + DMSO	2.82 ± 0.05^h^	2.98 ± 0.09^d^	45.28 ± 0.37^h^	12.26 ± 1.45^f^	16.74 ± 0.45^g^
CCl_4_	1.12 ± 0.06^a^	1.21 ± 0.10^a^	20.69 ± 0.39^a^	25.61 ± 0.32^a^	32.45 ± 0.19^a^
Sily + CCl_4_	2.55 ± 0.08^g^	2.67 ± 0.14^c^	38.96 ± 0.60^g^	15.45 ± 0.74^e^	19.48 ± 0.90^f^
HRR + CCl_4_	1.40 ± 0.04^b^	1.34 ± 0.06^a^	22.34 ± 0.42^b^	22.05 ± 0.80^b^	29.04 ± 0.45^b^
ERR + CCl_4_	1.45 ± 0.10^b^	1.33 ± 0.09^a^	22.31 ± 0.38^b^	23.14 ± 0.62^b^	28.92 ± 0.36^b^
CRR + CCl_4_	1.75 ± 0.11^c^	1.64 ± 0.10^b^	25.62 ± 0.71^c^	20.55 ± 0.70^c^	25.24 ± 0.74^c^
BRR + CCl_4_	2.30 ± 0.07^f^	2.26 ± 0.11^c^	33.61 ± 0.32^e^	17.90 ± 0.63^d^	23.51 ± 0.17^d^
MRR + CCl_4_	2.25 ± 0.06^e^	2.44 ± 0.12^c^	34.37 ± 0.25^f^	18.26 ± 0.56^d^	21.35 ± 0.78^e^
ARR + CCl_4_	2.02 ± 0.05^d^	2.04 ± 0.13^c^	28.55 ± 0.65^d^	20.31 ± 0.38^c^	25.37 ± 0.28^c^
HRR alone	2.80 ± 0.08^h^	2.93 ± 0.11^d^	45.22 ± 0.47^h^	12.11 ± 0.65^f^	16.73 ± 0.47^g^
ERR alone	2.78 ± 0.16^h^	2.88 ± 0.18^d^	45.53 ± 0.60^h^	10.53 ± 0.71^f^	17.03 ± 0.80^g^
CRR alone	2.99 ± 0.09^h^	3.00 ± 0.10^d^	45.48 ± 0.49^h^	13.06 ± 1.06^f^	16.61 ± 0.35^g^
BRR alone	2.75 ± 0.05^h^	3.18 ± 0.09^d^	45.78 ± 0.46^h^	9.76 ± 1.93^f^	16.74 ± 0.65^g^
MRR alone	2.98 ± 0.09^h^	3.11 ± 0.06^d^	46.05 ± 0.26^h^	10.45 ± 1.48^f^	14.63 ± 0.97^g^
ARR alone	2.93 ± 0.10^h^	3.08 ± 0.07^d^	46.13 ± 0.58^h^	12.17 ± 1.42^f^	15.47 ± 0.87^g^

Values are mean ± SD (06 number). Sily: Silymarin.

^a–h^(means with different letters) indicate significance at *P* < 0.05.

**Table 4:** Effects of various fractions of *R. hastatus* roots on tissue proteins and antioxidant enzyme concentrations.

Group	Protein (*µ*g/mg tissue)	CAT (U/min)	POD (U/min)	SOD (U/mg protein)	TBARS (nM/min/mg protein)	H_2_O_2_ (*μ*M/mL)
Control	2.27 ± 0.020^f^	4.80 ± 0.10^d^	14.31 ± 0.25^d^	4.33 ± 0.46^d^	2.30 ± 0.41^c^	1.89 ± 0.10^c^
Oil + DMSO	2.30 ± 0.010^f^	4.75 ± 0.15^d^	13.90 ± 0.20^d^	4.10 ± 0.28^d^	2.08 ± 0.33^c^	1.78 ± 0.12^c^
CCl_4_	1.05 ± 0.021^a^	2.10 ± 0.07^a^	6.67 ± 0.17^a^	1.23 ± 0.54^a^	6.52 ± 0.58^a^	3.54 ± 0.26^a^
Sily + CCl_4_	1.91 ± 0.023^e^	3.97 ± 0.06^c^	11.67 ± 0.44^c^	3.14 ± 0.43^c^	3.11 ± 0.80^b^	2.18 ± 0.31^b^
HRR + CCl_4_	1.42 ± 0.009^b^	2.92 ± 0.32^b^	9.09 ± 0.34^b^	1.96 ± 0.16^b^	6.00 ± 0.54^a^	3.40 ± 0.17^a^
ERR + CCl_4_	1.43 ± 0.010^b^	3.07 ± 0.20^b^	9.57 ± 0.21^b^	2.05 ± 0.21^b^	6.18 ± 0.33^a^	3.29 ± 0.11^a^
CRR + CCl_4_	1.50 ± 0.013^c^	3.63 ± 0.17^c^	10.13 ± 0.64^c^	2.38 ± 0.31^b^	5.03 ± 0.26^b^	2.89 ± 0.20^b^
BRR + CCl_4_	1.82 ± 0.085^e^	3.99 ± 0.15^c^	11.01 ± 0.24^c^	2.90 ± 0.15^c^	4.18 ± 0.92^b^	2.69 ± 0.31^b^
MRR + CCl_4_	1.78 ± 0.060^e^	4.00 ± 0.26^c^	11.61 ± 0.75^c^	3.32 ± 0.43^c^	3.65 ± 0.75^b^	2.27 ± 0.42^b^
ARR + CCl_4_	1.65 ± 0.014^d^	3.80 ± 0.10^c^	10.60 ± 0.55^c^	2.96 ± 0.18^c^	4.62 ± 0.71^b^	2.77 ± 0.25^b^
HRR alone	2.30 ± 0.011^f^	4.71 ± 0.12^d^	14.68 ± 0.62^d^	4.21 ± 0.50^d^	2.19 ± 0.74^c^	1.82 ± 0.34^c^
ERR alone	2.33 ± 0.019^f^	4.65 ± 0.24^d^	14.71 ± 0.71^d^	4.78 ± 0.36^d^	2.34 ± 0.82^c^	1.84 ± 0.57^c^
CRR alone	2.44 ± 0.010^f^	4.91 ± 0.27^d^	14.99 ± 0.53^d^	4.64 ± 0.45^d^	2.52 ± 0.67^c^	1.96 ± 0.77^c^
BRR alone	2.26 ± 0.009^f^	4.96 ± 0.41^d^	14.66 ± 0.45^d^	4.71 ± 0.33^d^	2.24 ± 0.63^c^	1.93 ± 0.61^c^
MRR alone	2.38 ± 0.028^f^	4.86 ± 0.21^d^	15.07 ± 0.23^d^	4.73 ± 0.21^d^	2.18 ± 0.64^c^	2.01 ± 0.49^c^
ARR alone	2.35 ± 0.012^f^	4.84 ± 0.15^d^	15.01 ± 0.10^d^	4.58 ± 0.26^d^	2.39 ± 0.45^c^	1.92 ± 0.70^c^

Values are mean ± SD (06 number). Sily: Silymarin.

^a–f^(means with different letters) indicate significance at *P* < 0.05.

**Table 5:** Effects of various fractions of *R. hastatus* roots on phase II antioxidant enzymes and DNA fragmentation.

Group	GST (nM/mg protein)	GPx (nM/mg protein)	GR (nM/mg protein)	GSH (*µ*M/g tissue)	QR (nM/mg protein)	Percent DNA injuries
Control	150.21 ± 4.11^g^	110.71 ± 3.23^e^	198.47 ± 4.72^h^	17.69 ± 1.11^e^	105.33 ± 1.34^g^	9.21 ± 1.30^e^
Oil + DMSO	146.34 ± 4.10^g^	104.56 ± 3.10^e^	190.69 ± 4.39^h^	15.40 ± 1.30^e^	107.12 ± 1.46^g^	8.38 ± 1.63^e^
CCl_4_	87.48 ± 3.45^a^	57.86 ± 2.67^a^	120.76 ± 3.02^a^	7.63 ± 0.75^a^	63.34 ± 1.01^a^	51.11 ± 2.02^a^
Sily + CCl_4_	122.22 ± 3.18^e^	94.62 ± 2.47^d^	158.61 ± 2.35^g^	13.73 ± 0.73^d^	91.54 ± 2.11^f^	22.43 ± 1.57^d^
HRR + CCl_4_	94.05 ± 2.56^b^	68.44 ± 2.37^b^	127.11 ± 2.34^b^	10.08 ± 0.39^b^	69.69 ± 1.72^b^	42.22 ± 1.27^b^
ERR + CCl_4_	95.67 ± 2.48^b^	70.37 ± 2.42^b^	127.51 ± 2.55^b^	10.25 ± 0.36^b^	71.47 ± 1.95^b^	35.33 ± 2.15^c^
CRR + CCl_4_	100.28 ± 2.27^c^	76.22 ± 2.71^c^	132.17 ± 2.33^c^	11.17 ± 0.28^c^	75.56 ± 1.38^c^	26.44 ± 1.73^d^
BRR + CCl_4_	119.16 ± 3.15^e^	80.51 ± 2.33^c^	144.43 ± 2.26^e^	12.19 ± 0.43^d^	83.24 ± 2.82^e^	25.53 ± 2.15^d^
MRR + CCl_4_	130.53 ± 2.34^f^	88.61 ± 2.20^c^	150.39 ± 2.41^f^	12.36 ± 0.15^d^	90.97 ± 2.61^f^	23.78 ± 1.23^d^
ARR + CCl_4_	108.26 ± 3.68^d^	85.47 ± 2.62^c^	139.40 ± 2.16^d^	11.53 ± 0.32^c^	79.87 ± 2.28^d^	25.73 ± 1.56^d^
HRR alone	149.67 ± 3.63^g^	107.34 ± 3.57^e^	192.22 ± 2.23^h^	16.22 ± 0.50^e^	108.34 ± 1.41^g^	7.66 ± 1.27^e^
ERR alone	155.87 ± 4.58^g^	106.48 ± 4.53^e^	201.47 ± 3.50^h^	18.37 ± 0.38^e^	100.44 ± 1.70^g^	7.22 ± 1.43^e^
CRR alone	148.51 ± 3.45^g^	109.38 ± 3.22^e^	191.71 ± 3.00^h^	15.30 ± 0.38^e^	109.34 ± 1.24^g^	7.80 ± 1.28^e^
BRR alone	156.63 ± 4.43^g^	107.68 ± 4.34^e^	195.45 ± 3.45^h^	17.04 ± 0.38^e^	108.45 ± 1.89^g^	8.34 ± 0.28^e^
MRR alone	156.52 ± 3.23^g^	111.51 ± 3.38^e^	200.10 ± 2.69^h^	18.10 ± 0.23^e^	110.71 ± 1.57^g^	8.23 ± 0.93^e^
ARR alone	157.77 ± 3.76^g^	102.57 ± 3.43^e^	197.53 ± 4.02^h^	15.37 ± 0.20^e^	109.34 ± 1.45^g^	6.78 ± 1.73^e^

Values are mean ± SD (06 number). Sily: Silymarin.

^a–h^(means with different letters) indicate significance at *P* < 0.05.
